# Personalized warning signals for depressive relapse: A qualitative study

**DOI:** 10.1192/j.eurpsy.2021.889

**Published:** 2021-08-13

**Authors:** C. Slofstra, S. Castelein, A. Pieper, G. Dijkstra, R. Hoenders, S. Booij

**Affiliations:** 1 Lentis Research, Lentis, Groningen, Netherlands; 2 Center For Integrative Psychiatry, Lentis, Groningen, Netherlands

**Keywords:** Relapse prevention, Depression, Personalized warning signals, Personalized mental health

## Abstract

**Introduction:**

An important aspect of depression relapse prevention programs is identifying personalized warning signals (PWS). These PWS are typically defined as depressive symptoms. Yet, no study has investigated to what extend PWS fit within the diagnostic classification framework, and how this compares to a more transdiagnostic, integrative approach towards depression.

**Objectives:**

To examine how well PWS reflect depressive symptoms, describe the remaining PWS, and examine how well PWS can be assigned to domains of an existing transdiagnostic and integrative framework, the positive health concept.

**Methods:**

162 PWS of 66 individuals with a history of depression were labeled as one or more symptoms of depression or to a residual category. The same process was repeated for labeling the domains of the positive health model. Labeling was done by three independent reviewers (inter-rater percent agreement: symptoms: 0.83 & positive health domains: 0.73). Disagreements were resolved by discussion.

**Results:**

The three most commonly reported depressive symptoms were insomnia/hypersomnia, anhedonia and fatigue/loss of energy. However, sixty-five percent of the PWS were not depressive symptoms, but other symptoms (e.g. irritability, rumination) or aspects of functioning (e.g. withdrawing, managing time). The positive health domains captured all the PWS. However, 44% of PWS were labeled as multiple positive health domains, whereas labeling as symptoms of depression resulted in almost no such overlap.

**Conclusions:**

A more transdiagnostic and integrative approach seems necessary to capture PWS. Depending on one’s purpose, one may consider expanding the definition with other symptoms and aspects of functioning, or using the positive health concept.

